# Understanding the role of intermolecular interactions between lissoclimides and the eukaryotic ribosome

**DOI:** 10.1093/nar/gkz053

**Published:** 2019-02-13

**Authors:** Simone Pellegrino, Mélanie Meyer, Zef A Könst, Mikael Holm, Vamsee K Voora, Daniya Kashinskaya, Camila Zanette, David L Mobley, Gulnara Yusupova, Chris D Vanderwal, Scott C Blanchard, Marat Yusupov

**Affiliations:** 1 Institut de Génétique et de Biologie Moléculaire et Cellulaire (IGBMC), INSERM U964, CNRS UMR7104, Université de Strasbourg, 67404 Illkirch, France; 2 Department of Chemistry, University of California, 1102 Natural Sciences II, Irvine, CA 92697-2025, USA; 3 Department of Physiology and Biophysics, Weill Cornell Medicine, New York, NY, USA; 4 Institute of Fundamental Medicine and Biology, Kazan Federal University, Kazan 420008, Russia; 5 Department of Pharmaceutical Sciences, University of California, Irvine, CA 91010-92697, USA; 6 Tri-Institutional PhD Training Program in Chemical Biology, Weill Cornell Medicine, Rockefeller University, Memorial Sloan-Kettering Cancer Center, New York, NY 10065, USA

## Abstract

Natural products that target the eukaryotic ribosome are promising therapeutics to treat a variety of cancers. It is therefore essential to determine their molecular mechanism of action to fully understand their mode of interaction with the target and to inform the development of new synthetic compounds with improved potency and reduced cytotoxicity. Toward this goal, we have previously established a short synthesis pathway that grants access to multiple congeners of the lissoclimide family. Here we present the X-ray co-crystal structure at 3.1 Å resolution of C45, a potent congener with two A-ring chlorine-bearing stereogenic centers with ‘unnatural’ configurations, with the yeast 80S ribosome, intermolecular interaction energies of the C45/ribosome complex, and single-molecule FRET data quantifying the impact of C45 on both human and yeast ribosomes. Together, these data provide new insights into the role of unusual non-covalent halogen bonding interactions involved in the binding of this synthetic compound to the 80S ribosome.

## INTRODUCTION

**Figure 1.**
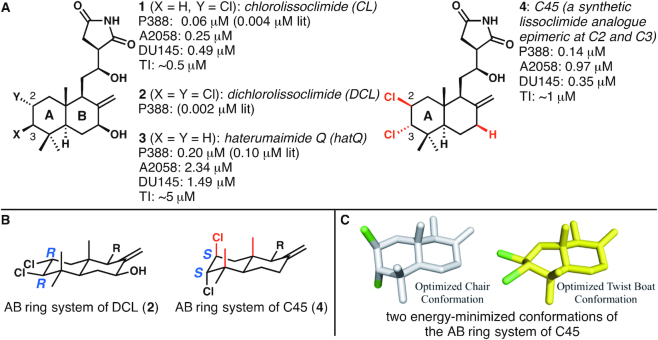
The structures of natural lissoclimide compounds chlorolissoclimide, dichlorolissoclimide, haterumaimide Q and synthetic analogue C45. (**A**) All data shown are IC_50_ values against the cell lines shown (P388: murine leukemia; A2058: aggressive melanoma; DU145: aggressive prostate cancer), except for TI, which represents translation inhibition data. Literature values of IC_50_s against P388 (indicated by ‘lit’) are taken from reference (12), and all other data are taken from reference (3). (**B**) A comparison of the configurations of the C2 and C3 chloride-bearing stereogenic centers in the natural product DCL and the synthetic analogue C45. (C) Energy-minimized conformations of the AB ring system of C45: the first has the A ring in a chair conformation, and four substituents axial; the second has the A ring in a twist-boat conformation, thus relieving multiple 1,3-diaxial interactions.

The ribosome is the central player in protein biosynthesis in all living organisms. In eukaryotic species, this massive (∼4.3 MDa) ribonucleoprotein assembly, which is composed of four types of ribosomal RNA (rRNA) and ∼80 ribosomal proteins, has a significant role in regulation of cell growth. For this reason, it is not surprising that the ribosome represents a useful target for the inhibition of proliferative cancers, often characterized by dysregulated protein synthesis (1–5). Deep understanding of the capacity of small-molecule drugs to target the eukaryotic 80S ribosome and arrest the growth of tumor cells is of paramount importance in this line of research (2–7). The lissoclimide family of compounds has been shown to have cytotoxic activity towards a variety of carcinoma cell lines, with some members reaching subnanomolar potencies (half-maximum inhibitory concentration (IC_50_) values). Chlorolissoclimide (CL) and dichlorolissoclimide (DCL) (**1** and **2**, respectively, Figure 1A) in particular were characterized as inhibitors of eukaryotic protein synthesis. Previous biochemical experiments suggested that these compounds specifically act to hinder the elongation phase of translation, preventing P-site tRNA from proceeding further into the ribosomal E-site, thereby blocking cellular functions (8).

To validate this hypothesis, the crystal structure of CL in complex with the eukaryotic 80S ribosome was recently determined, elucidating at atomic resolution the binding mode of CL (3). The target on the ribosome is indeed the E-site of the large ribosomal subunit (LSU), similar to the glutarimide antibiotics cycloheximide (CHX) and lactimidomycin (2). Several interesting features of the binding of CL to the ribosome were discovered; the most unusual was the halogen–π interaction between the C2 chlorine and the rRNA residue G2794 of the 25S rRNA, which leads to a substantial stabilization of CL within the E-site (with an energetic benefit of ∼1.8 kcal mol^−1^ (3)). Surprisingly, given the large spectrum of different ribosome inhibitors, this particular type of interaction has only been disclosed two other times. In one case, Romo, Yusupov, Green, Liu *et al.* recently reported that a bromide located on the pyrrole portion of the translation inhibitor agelastatin A apparently engages in a dispersion interaction with a proximal uracil (4). Llano-Sotelo *et al.* have instead reported the halogen–π interaction established between the fluorine atom, located in position C2 of the 14-atom lactone ring, of the aminoglycoside CEM-101 and the rRNA residue C2611 in bacteria (9). Related arrangements between an alkyl fluoride in aminoglycosides and rRNA bases were reported earlier by Hanessian and co-workers (10).

To further expand the search for lissoclimides with increased potencies with regard to protein synthesis inhibition and cancer cells cytotoxicity, semi-synthesis and analogue-oriented synthesis strategies were specifically designed to produce CL and its synthetic congeners (3,11). In particular, a recently developed analogue-oriented synthesis allowed us to access several analogues that were tested against P388 murine leukemia, aggressive melanoma (A2058) and prostate cancer (DU145) cell lines (3). The second most potent compound of the congeners created with the aforementioned chemical synthesis is called C45 (4, Figure 1A), which was made in the course of attempts to make DCL and other lissoclimide congeners bearing both C2 and C3 chlorides; DCL was known to be slightly more cytotoxic toward P388 cell lines than CL (12). To date, all of the chlorinated lissoclimides isolated from nature bear their chlorine atoms with the same configurations (for DCL [**2**], C2 = *R*, C3 = *R*). In the presumed lowest energy (chair) conformation of the A ring in DCL (Figure 1B), the two chlorine atoms were expected to each reside in an equatorial orientation. In contrast, C45 has each chloride installed with the configurations (C2 = *S*, C3 = *S*) that are opposite to those seen in the natural products; therefore, the nearly equal cytotoxicity (140 nM IC_50_) and translation inhibition activity (∼0.5 μM IC_50_ value) as compared with CL (60 nM and 0.5 μM, respectively) (3) was particularly surprising. It seemed inconceivable that C45 would fit in the ribosome binding pocket with two epimeric chlorine-bearing stereogenic centers, because these chlorines would be expected to be oriented axially in a chair-like conformation (Figure 1B). However, molecular docking experiments conducted previously suggested that C45 might make a chair to twist-boat conformational change, thus alleviating several 1,3-diaxial interactions, to display each (nominally axial) chloride atom in a more ‘equatorial’ orientation (Figure 1C) and place each of them in proximity to the faces of guanine nucleobases (3). Put another way, the docking experiment—which does not take into account attractive dispersion interactions—suggested the possibility that each chlorine may make a halogen–π interaction with guanine residues within the E-site pocket. Therefore, C45 represents a good candidate to deepen our knowledge on this unusual type of interaction and on the mode of binding of lissoclimides in general.

Here, we present the 3.1 Å resolution X-ray structure of C45 in complex with the yeast 80S ribosome, together with single-molecule fluorescence resonance energy transfer (smFRET) imaging studies, and intermolecular interaction energies obtained by density functional theory calculations. The totality of these data sets permit us to dissect the molecular mechanism by which this member of the lissoclimide family inhibits protein synthesis in eukaryotes, showing the unique binding properties of halogenated compounds holding pharmacological potential.

## MATERIALS AND METHODS

### Ribosome purification, complex formation, crystallization and crystal treatment

80S ribosomes from the yeast *Saccharomyces cerevisiae* were purified as described previously (2,13). The C45/80S complex was formed in 5.5 mM Tris-acetate at pH 7.0, 27 mM K(OAc) at pH 7.2, 5.5 mM NH_4_(OAc), 2 mM Mg(OAc)_2_, 1.3 mM DTT by incubation of 80S ribosomes (1.515 μM) with 30-fold molar excess of C45 (45 μM) for 15 min at 30 °C. Crystals were grown at 4 °C by hanging-drop vapor diffusion based on the previously described protocol (2). Crystals were then treated as in (2) with a constant concentration of C45 of 45 μM and an increased glycerol concentration to 20% in all intermediate solutions.

### Data collection, processing and structure determination

Data were collected at SOLEIL, PROXIMA1 beamline at cryogenic temperature (100 K), using a Pilatus-6M detector. Low dose data were collected at a wavelength (λ) of 1.148 at high redundancy and then processed and scaled using the XDS suite (14). The resulting file was converted into mtz format (XDSCONV program) and then subjected to a first cycle of rigid body refinement in phenix.refine (PHENIX suite, (15)). The vacant *S. cerevisiae* 80S ribosome (PDB ID: 4V88) was used as search model, with each chain refined as a single rigid body. Difference density map (*F*_obs_ – *F*_calc_) was then manually inspected for the presence of the inhibitor, which was then placed into the density. Electron density for C45 compound was observed only at a single binding site. Drawing of the chemical structure was performed using MarvinSketch suite (ChemAxon, http://www.chemaxon.com/). Coordinates and restraints of the ligand were generated by submitting the 3D coordinates previously generated to the GradeWebServer (http://grade.globalphasing.org). Ligand fitting and remodeling of the LSU E-site binding pocket was performed manually using Coot (16). Further cycles of coordinates and individual isotropic *B*-factor refinement, taking one *B*-factor per residue, were performed using phenix.refine (15), yielding the crystallographic statistics presented in Table 1. Structure validation was performed using Molprobity (17). Ramachandran plot analysis result in 86.61% favored, 12.52% allowed and 0.84% outliers. Figures of the structure were prepared using PyMOL 1.7.4 (Schrödinger, https://pymol.org/2/).

**Table 1.** Data collection and refinement statistics

	C45/80S complex
**Data collection**	
Space group	*P*2_1_
Cell dimensions	
*a, b, c* (Å)	303.77, 287.95, 435.45
*α, β, γ* (°)	90.00, 98.96, 90.00
Resolution (Å)	55.00–3.10 (3.20–3.10)^a^
*R*_meas_(%)	42.2 (277.6)
*I*/σ*I*	7.65 (1.20)
CC_1/2_ (%)	99.2 (50.4)
Completeness (%)	100 (100)
Redundancy	16.43 (15.69)
**Refinement**	
Resolution (Å)	54.71–3.10
No. reflections	1 332 618
*R*_work/_ *R*_free_^b^	0.2167/0.2645
No. atoms	
Protein	178 254
RNA	222 470
Ions/ligands	8946
*B*-factors	
Protein	61.38
RNA	58.62
Ions/ligands	82.20
R.m.s deviations	
Bond lengths (Å)	0.010
Bond angles (°)	1.354

Number of crystals used: 11.

^a^Values in parentheses are for highest-resolution shell.

^b^Rfree flags (2% of the total number of reflections) were taken from the CL/80S complex dataset (PDB ID: 5TBW).

### Computational analysis

**Figure 2.**
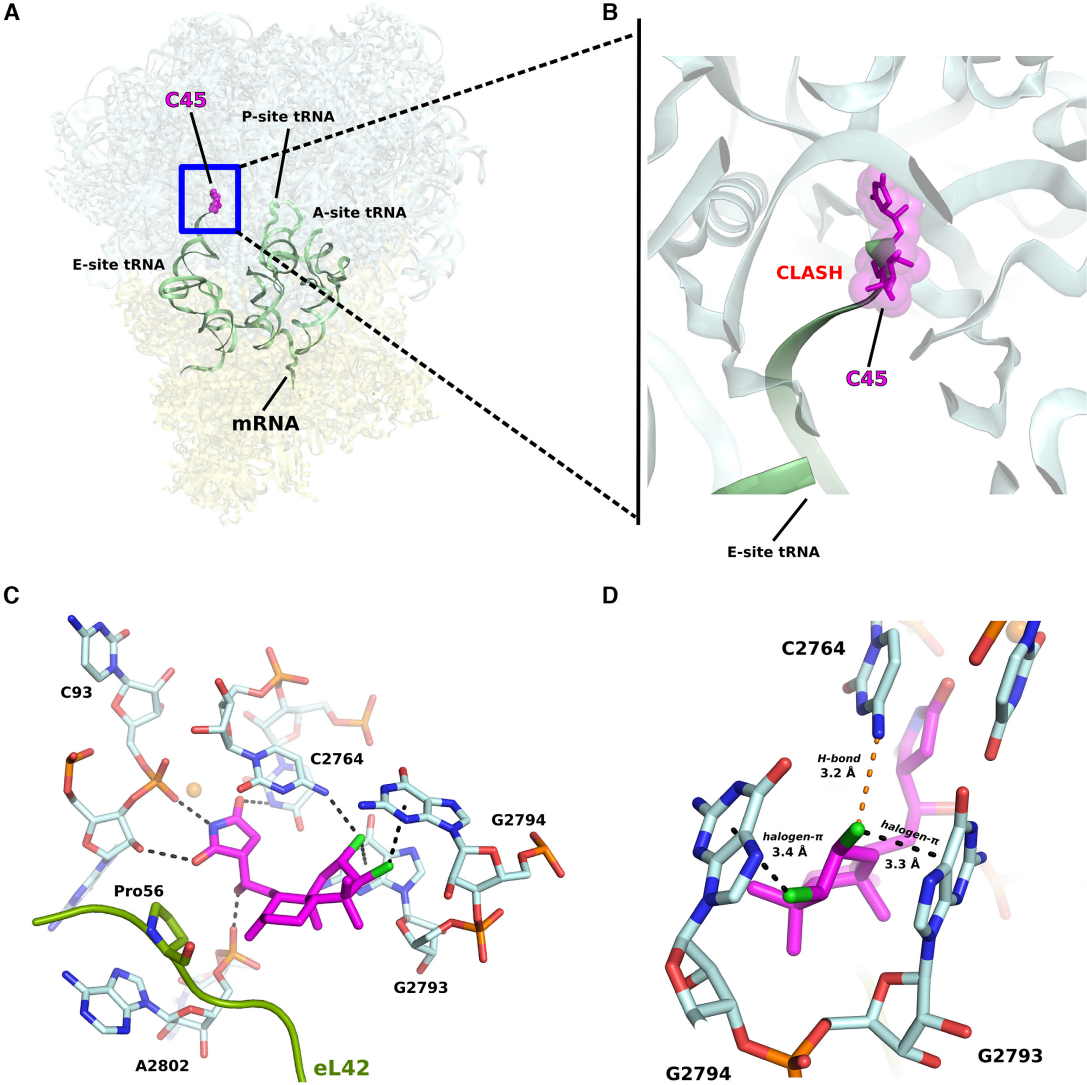
C45 binds to the E-site of the LSU on the eukaryotic 80S ribosome. (**A**) Overall representation of binding pose of C45 within its target. For clarity tRNAs and mRNA are shown as outline, to mimic an actively translating ribosome (tRNAs and mRNA were taken from PDB: 4V6F), although they are not present in the present structure. (**B**) Zoom into the E-site binding pocket which highlights how the actual positioning of C45 would sterically clash with the incoming deacylated tRNA during elongation. (**C**) Close-up view of C45 binding pocket, showing the interactions that C45 establishes with the 25S rRNA residues. In the case of C45, there is no interaction with the eukaryotic specific ribosomal protein eL42, as previously reported for CL (PDB: 5TBW). (D) Detailed view of the halogen–π interactions occurring between the chlorine groups onto the decalin ring of C45 and the 25S rRNA residues G2793 and G2794. Distance is also reported for the H-bond between the chlorine in position C2 of C45 with C2764.

The initial structures for the reduced models (Figure 2), obtained from the X-ray crystal structures, were geometry optimized for the H-atom positions using the D3 (18) dispersion-corrected Becke-Lee-Yang-Parr density functional approximation (19,20). For the geometry optimization, at least m5 grids (21) and def2-TZVPP (22) basis sets were used. An energy convergence criterion of 10^−7^ a.u. and a gradient convergence of 10^−3^ a.u. were used throughout. All calculations were carried out using TURBOMOLE V7.2 (23).

### Single-molecule smFRET

All smFRET experiments were conducted at 23 °C in human polymix buffer (50 mM Tris pH 7.5, 5 mM MgCl_2_, 50 mM NH_4_Cl, 2 mM spermidine, 5 mM putrescine) containing a mixture of triplet-state quenchers (1 mM Trolox, 1 mM 4-nitrobenzyl alcohol (NBA), 1 mM cyclooctatetraene (COT)) and an enzymatic oxygen scavenging system (2 μM 3,4-dihydroxybenzoic acid (PCA), 0.02 units/ml protocatechuate 3,4-dioxygenase (PCD)). Surface-immobilized human or yeast ribosome pre-translocation complexes were prepared as in (24). Briefly, eEF1A ternary complexes containing Cy5-labeled Phe-tRNA^Phe^ were delivered to surface-immobilized 80S initiation complexes programmed with P-site Cy3-labeled Met-tRNA^fMet^ and a short mRNA displaying a UUU codon in the A site. Ternary complex was washed from the flow cell with human polymix buffer after 30 s of incubation, leading to stoichiometric formation of pre-translocation ribosomes containing Cy3-labeled tRNA^fMet^ in the P site and Cy5-labeled Met-Phe-tRNA^Phe^ in the A site. The drugs were diluted in human polymix buffer, manually injected into the flow cell and allowed to equilibrate with the ribosome before data acquisition. To ensure that drug binding had equilibrated several movies were recorded over the span of ∼10 min and compared.

smFRET data was recorded using a home-built total internal reflection based fluorescence microscope (25) at ∼0.1 kW/cm^2^ laser (532 nm) illumination at a time resolution of 40 ms. Donor and acceptor fluorescence intensities were extracted from the recorded movies and FRET efficiency traces were calculated. FRET traces were selected for further analysis according to the following criteria: a single catastrophic photobleaching event, at least 8:1 signal/background-noise ratio and 6:1 signal/signal-noise ratio, less than four donor-fluorophore blinking events, a correlation coefficient between donor and acceptor <0.5 and a lifetime of at least 50 frames (2 s at 40 ms time resolution) in any FRET state ≥0.15.

smFRET traces were analyzed using hidden Markov model idealization methods as implemented in the SPARTAN software package (25). In all idealizations, transitions between all states were allowed in a three state model of the human or yeast pre-translocation ribosome (FRET values 0.26 ± 0.07, 0.44 ± 0.06 and 0.71 ± 0.05 for human, and 0.22 ± 0.05, 0.40 ± 0.06 and 0.65 ± 0.04 for yeast). To estimate equilibrium binding constants for the three drugs the fraction of ribosomes occupying the Classical state (FRET 0.71 ± 0.05) was plotted against drug concentration and the following equation was fitted to the data.

\begin{equation*}\;{f_c} = \frac{{1 + \frac{{\left[ I \right]}}{{{K_I}}}}}{{1 + {K_0} + \frac{{\left[ I \right]}}{{{K_I}}}\left( {1 + \frac{1}{{{K_2}}}} \right)}}\;\end{equation*}

Here, *f*_*c*_ is the fraction of classical state ribosomes, *K_I_* is the drug dissociation constant, *K*_0_ is the equilibrium constant for the classical to hybrid transition on the drug-free ribosome, *K*_2_ is the equilibrium constant for the classical to hybrid transition on the drug-bound ribosome and [*I*] is the drug concentration.

## RESULTS

### Structural studies reveal the binding mode of C45 to the eukaryotic 80S ribosome

We solved the X-ray crystal structure of the *S. cerevisiae* 80S ribosome in complex with a synthetic sample of the inhibitor C45 at a maximal resolution of 3.1 Å (Figure 2A, B, Table 1). As shown for its congener, CL (3), C45 binds within the large ribosomal subunit (LSU) E-site pocket (Figure 2). The molecular mechanism by which C45 inhibits protein synthesis in eukaryotes is the same as proposed for CL (3,8). Its binding impairs the entrance of the CCA-end of the tRNA into the E-site pocket during the elongation phase of protein synthesis.

To solve the present structure we used the vacant 80S ribosome (PDB (Protein Data Bank) entry: 4V88 (13)) as reference model. The resulting difference density map (*F*_obs_ – *F*_calc_) (Supplementary Figure S1) allowed us to unambiguously fit C45 within the binding pocket, revealing its mode of binding. As previously predicted by docking studies (3), C45 adopts a twist-boat conformation when binding within the E-site pocket of the LSU. We analyzed in more detail the binding mode of C45 and compared it to that of its congener, CL, first of all with regard to the unique contact that the latter forms with the eukaryotic specific ribosomal protein eL42 (3). As C45 lacks the hydroxyl group at position C7 in CL, it cannot hydrogen bond with the Pro56 of eL42. Structural superposition of the E-site binding pocket of the CL/80S complex with the same region of the present structure (RMSD: 0.187 Å) further revealed a different overall positioning of the decalin ring of C45 within the binding pocket. Although the succinimide moiety creates the same set of hydrogen bonds with the 25S rRNA (Figure 2C, D and Supplementary Figure S2A, B), we observed that it is slightly tilted toward the rRNA residues G92, C93 and A2802 (approximately 0.6 Å), most likely to allow the decalin ring, which in C45 adopts a twist-boat conformation, to be accommodated within the E-site binding pocket. Additionally, we observed that C45 forms two halogen–π interactions, with a face-on geometry, with two consecutive guanosines, G2793 and G2794, as previously predicted by docking studies (3). Both chlorine atoms are positioned 3.4 Å away from the centers of the aromatic rings of the rRNA residues G2793 and G2794 involved in binding. We further noticed that the chlorine atom in position C2 approaches within 3.2 Å the rRNA residue C2764, while in the case of CL, this distance was observed to be 3.6 Å (Supplementary Figure S2B).

### Dispersion interaction calculation confirms the unique structural features of C45

**Figure 3.**
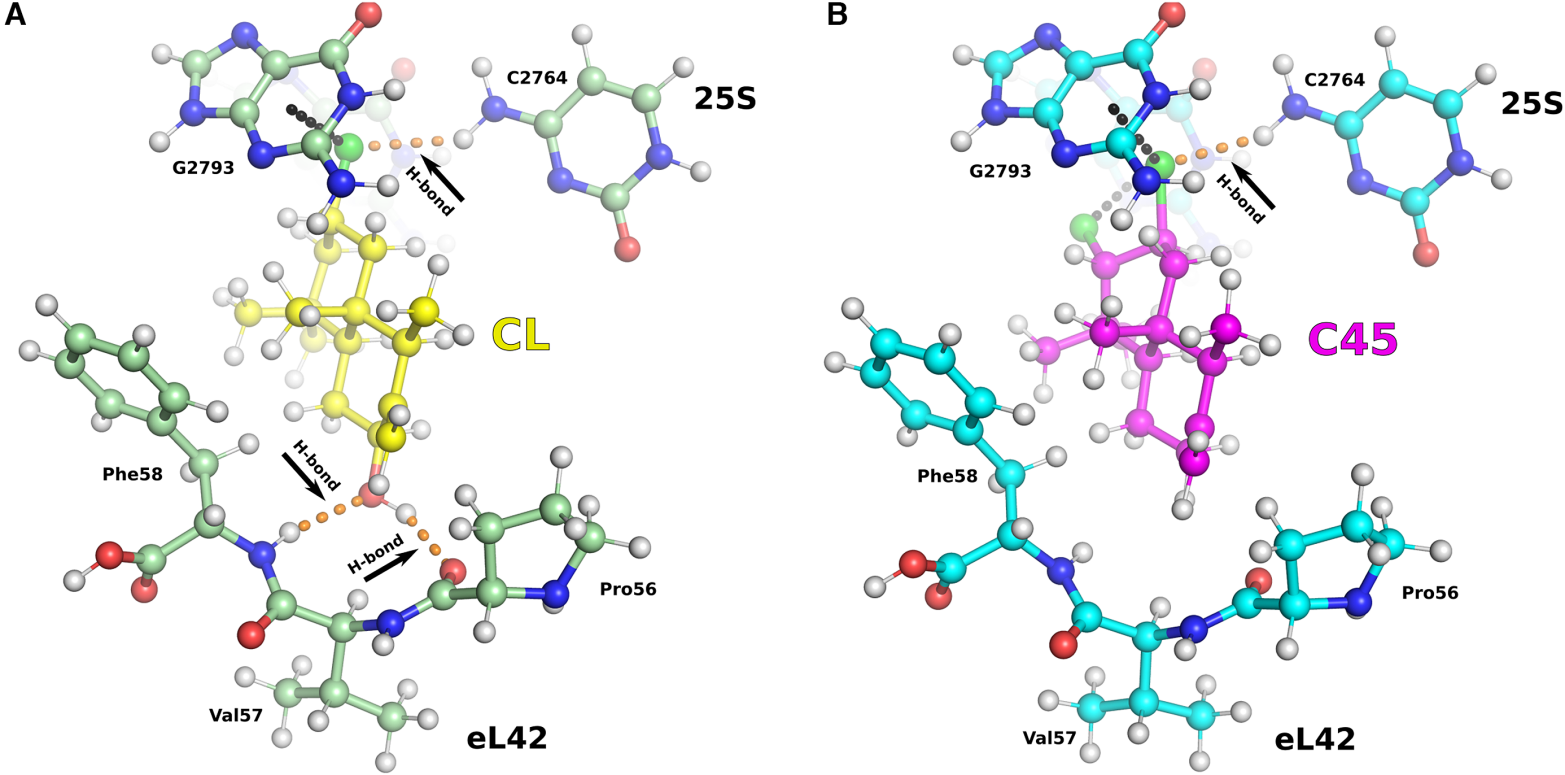
The binding mode for C45/RNA complex dissected by computational analysis. The minimized model consists of the lissoclimide compounds, the rRNA residues of the E-site pocket (reduced to C2764, G2793 and G2794) and the PVF chain (Pro-Val-Phe) of eL42. The O atoms are shown in red, Cl in green, N in blue and H in white. Calculated interaction energies (Table 2) pointed out the importance of the H-bonds formed between CL and eL42 ribosomal protein (**A**) in providing higher potency compared to C45, where these interactions are lost (**B**). However an additional halogen–π interaction and a shorter distance interaction with C2764 of the 25S rRNA might partially compensate for the absence of H-bonding.

Visual analysis of the crystal structures of C45 and CL (PDB: 5TBW) in complex with the 25S rRNA of the 80S ribosome (C45/RNA and CL/RNA complexes hereafter) revealed that there are at least three different bases (two guanines and one cytosine) of the rRNA pocket that are non-covalently interacting with the A-ring of the lissoclimide compounds. The main mode of interaction between the compounds and guanine bases is through Cl-π interactions, while the interaction between the compounds and the cytosine base is a Cl$ \cdots$H–N hydrogen-bonding interaction. These involve halogen$ \cdots$π or halogen$ \cdots$H-N interactions as indicated in Figure 3. For CL, we noticed additional O$ \cdots$H–N and O$ \cdots$H–O hydrogen-bonding interactions with the peptide backbone of residues Pro56 and Phe58 of ribosomal protein eL42. To further understand these interactions, we considered a reduced model: the rRNA residues of the pocket were minimized to guanines G2793 and G2794, cytosine C2764 (the nearest residues to CL and C45) as well as a small peptide chain consisting of Pro-Val-Phe (from ribosomal protein eL42). Further, the CL and C45 compounds were modeled as substituted decalin rings (Figure 3). The structures for these model systems were obtained from the experimentally determined X-ray structures (PDB ID: 5TBW and present work) followed by geometry-optimization of the positions of hydrogen atoms. At the optimized geometries, the binding energies were evaluated using the Becke-Lee-Yang-Parr (BLYP) density functional approximation (19,20). Dispersion effects were included via the D3 method (18).

In Table 2, we report the individual (with C2764, G2793/94 and the peptide Pro-Val-Phe considered as independent entities) interaction energies and the overall (all of the above considered together) net interaction energy of various residues within the E-site involved in binding with C45 and CL. The sum of the energies for the interactions that we studied was larger for CL than for C45 by 12.23 kcal/mol. This simplified analysis might suggest that CL should be many orders of magnitude better as a ribosome binder and therefore as an inhibitor of translation; however, it is critical to note that we are examining only selected interactions in isolation. Clearly, other potential factors must be contributing to binding affinity, by either destabilizing the binding of CL or by stabilizing that of C45. This would explain why the results of our experiments comparing CL and C45 for ribosome binding, translation inhibition, and cytotoxicity do not differ greatly. The net binding energies for C45 and CL to the E-site rRNA residues, represented by the two guanine (G2793 and G2794) and cytosine (C2764) bases, are very similar. Most of the energy difference shown in the Table 2 arises from two hydrogen bonds between the C7-hydroxyl group in the decalin ring of CL, which are missing in C45, and the backbone ribosomal protein eL42. The energy of desolvation of the C7-hydroxyl group in C45 (estimated to be ca. 9 kcal/mol (26)) would, by itself, significantly offset the positive gains from these hydrogen bonds. In the absence of desolvation effects, the interactions energies provide a simple analysis of the key, readily identifiable intermolecular interactions between the small molecules and the ribosome, and are not meant to quantify expected binding affinities, which are examined by experiment later in this report.

**Table 2.** Binding energies (in kcal/mol) of the representative complexes of C45 and CL with various components of the E-site pocket

	C45	CL
PVF	–5.86	–18.07
Cyto	–5.45	–5.17
Gua	–8.42	–8.68
PVF-Cyto-Gua	–19.26	–31.49

PVF denotes the Pro-Val-Phe peptide chain of eL42, Gua denotes the two guanine (G2793 and G2794) bases, and Cyto denotes the cytosine (C2764) base of the 25S rRNA. The binding energies, shown here, do not include deformation and desolvation energies.

### Experimental binding affinities of CL and C45 estimated by smFRET

**Figure 4.**
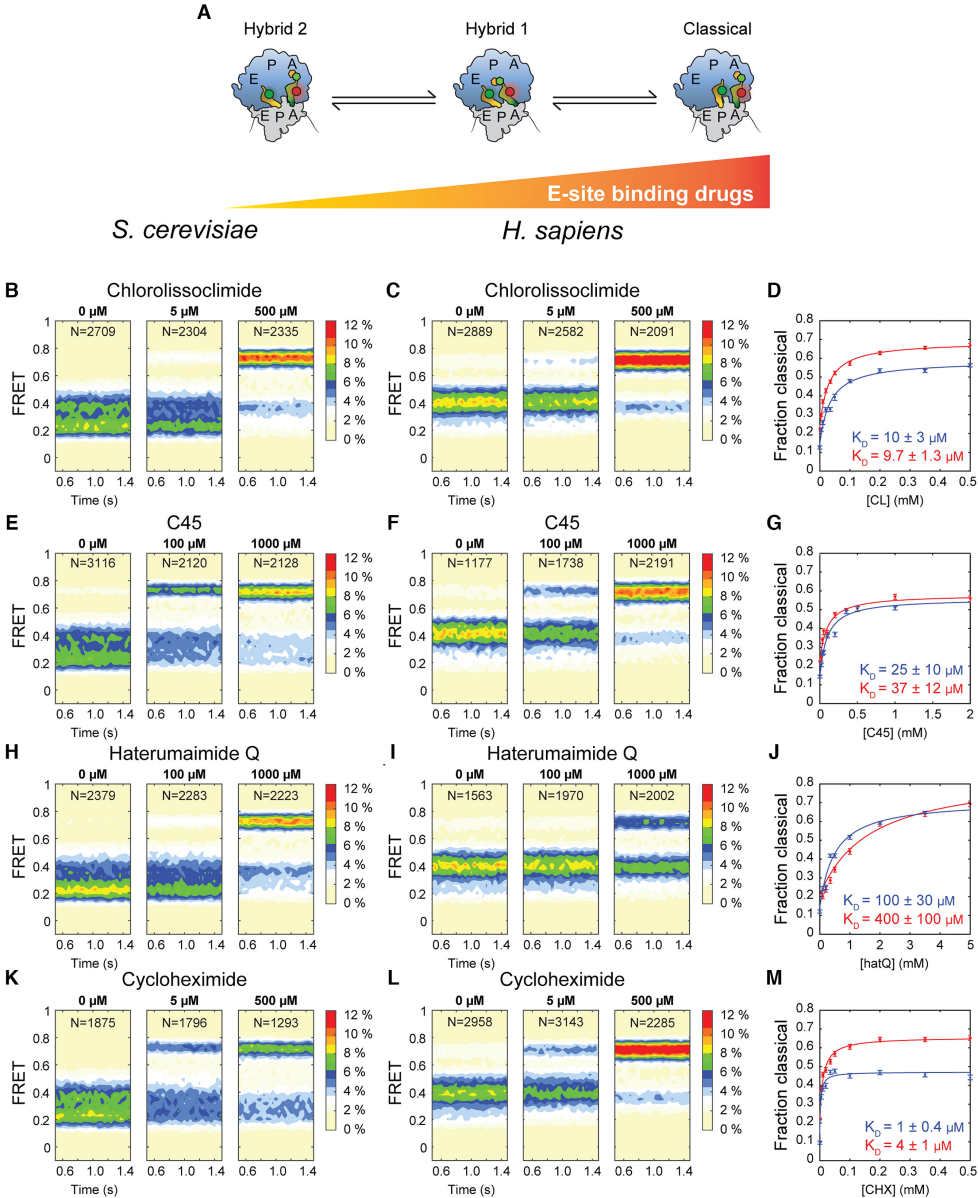
smFRET measurements of drug titrations on the yeast and human pre-translocation ribosome. Ribosomes containing P-site bound Cy3-tRNA^fMet^ and A-site bound Met-Phe-Cy5-tRNA^Phe^ were imaged at different drug concentrations and FRET between the two bound tRNA molecules was recorded. The ribosomes occupied three distinct FRET states that correspond to the Hybrid 2, Hybrid 1 and Classical tRNA conformations. (**A**) Schematic representation of the experiment: as the concentration of E-site binding drugs increases the ribosome increasingly favors classical over hybrid tRNA states. (B/C) Population FRET histograms of yeast and human pre-translocation ribosomes at the indicated concentrations of CL. *N* indicates the number of individual FRET traces used to construct the histograms. (**D**) The fraction of ribosomes in the high-FRET classical tRNA conformation as a function of CL concentration, estimated from data as in B and C, where blue data points indicate yeast and red data points indicate human. The solid lines represent fits of Eq. 1 to the data (Materials and methods), the blue K_D_ quantifies the affinity for the yeast ribosome and the red that for the human ribosome. (**E, F**) Population FRET histograms of yeast and human pre-translocation ribosomes at the indicated concentrations of C45. (**G**) The fraction of ribosomes in the high-FRET classical tRNA conformation as a function of CL concentration, estimated from data as in E and F. (**H, I**) Population FRET histograms of yeast and human pre-translocation ribosomes at the indicated concentrations of hatQ. (**J**) The fraction of ribosomes in the high-FRET classical tRNA conformation as a function of hatQ concentration, estimated from data as in H and I. (**K, L**) Population FRET histograms of yeast and human pre-translocation ribosomes at the indicated concentrations of CHX. (**M**) The fraction of ribosomes in the high-FRET classical tRNA conformation as a function of CHX concentration, estimated from data as in K and L. All error bars represent SEM.

To provide molecular insights into the impact of C45 and two related compounds, CL and hatQ (Figure 1), on functional *S. cerevisiae* 80S ribosome complexes (24) and experimentally determine their binding affinities, we employed single-molecule fluorescence resonance energy transfer (smFRET) imaging. As previously described for the human ribosome (24), we assembled yeast ribosome pre-translocation complexes by delivering Cy5-labeled Phe-tRNA^Phe^ in ternary complex with eEF1A and GTP to surface-immobilized ribosomes programmed with P-site bound Cy3 labeled Met-tRNA^fMet^ and a short mRNA displaying a UUU codon in the A-site. Using this system, we quantified FRET between adjacently bound, labeled-tRNAs within single pre-translocation complexes in the absence and presence of CL, C45 and hatQ to quantify their impacts on tRNA dynamics and position within the ribosome (24,27). In the absence of drug, the yeast pre-translocation ribosome occupied three, interconverting conformations with FRET efficiencies of 0.65 ± 0.04, 0.40 ± 0.06 and 0.22 ± 0.05, identified as the Classical, Hybrid-1 and Hybrid-2 states, respectively (24). Consistent with a vacant E-site, and like the mammalian ribosome, the yeast pre-translocation complex exhibited a strong preference for the hybrid over classical tRNA states (Figure 4). Unlike the previously characterized human ribosome, the yeast pre-translocation complex favored the Hybrid-2 state over the Hybrid-1 state. Titration of the E-site binding drugs shifted the equilibrium away from hybrid tRNA states towards classical conformations in a drug concentration-dependent manner (Figure 4). To more readily compare with the previously published cancer cell cytotoxic effects (24) we also carried out identical experiments on human pre-translocation complexes (Figure 4). In the absence of drugs the human pre-translocation complex also occupied three interconverting conformations with FRET efficiencies of 0.71 ± 0.05, 0.44 ± 0.06 and 0.26 ± 0.07, again identified as the Classical (A/A, P/P), Hybrid-1 (A/P, P/E) and Hybrid-2 (A/A, P/E) tRNA states, and it responded analogously to titration with the E-site binding drugs.

This effect is consistent with the suggested mode of action of the drugs: binding to the large subunit E-site, which is expected to block entry of the 3′-CCA end of P-site tRNA. Notably, both the human and yeast pre-translocation complexes were able to transition to lower-FRET, hybrid-like conformations, even at saturating drug concentrations (Supplementary Figure S3) indicating that such tRNA conformations are accessible even when the 3′-CCA end is unable to enter the large subunit E site on drug-bound ribosomes. Analogous transitions to hybrid-like (P/E*) positions have also recently been reported in bacterial systems when the P-site is occupied by peptidyl-tRNA bearing short nascent polypeptide chains (28–31).

By quantifying the shift in the distribution between Classical and Hybrid tRNA states during the drug titrations, we determined apparent dissociation constants for the three lissoclimide congeners to both yeast, with values of 10 ± 3 μM for CL, 25 ± 9 μM for C45 and 100 ± 30 μM for hatQ (Figure 4) and human, with values of 9.7 ± 1.3 μM for CL, 37 ± 12 μM for C45 and 400 ± 100 μM for hatQ (Figure 4), ribosomes. For comparison, we also determined the apparent dissociation constant for cycloheximide (CHX), a closely related, well-characterized compound, as 1 ± 0.4 μM to the yeast and 4 ± 1 μM to the human ribosome, respectively (Figure 4). Dissociation rate constants for the three congeners could not be accurately estimated due to either very long (CL, C45) or very short (hatQ) residence time on the ribosome.

## DISCUSSION

Compounds from the lissoclimide family have been shown to possess potential as therapeutics to treat cancers. CL has previously been characterized (3) and shown to be a potent anti-cancer agent against a broad variety of cancer cell lines. Thanks to the development of an efficient chemical synthesis, a large number of congeners of the lissoclimide family are now available and have already been characterized with respect to their cell growth inhibition activity. We decided to investigate more deeply the properties of a specific congener, C45, in terms of its binding mode to the eukaryotic 80S ribosome. We were interested in C45 for two reasons: (i) it is the second most potent cancer cell growth inhibitor of the synthetic compounds that we have studied (3) and (ii) it bears a second Cl atom in its decalin ring that was predicted, by docking experiments, to form an additional halogen–π interaction with its target. It was previously anticipated, by *ab initio* calculations, that the B ring of C45 might adopt a twist-boat conformation that reorients the chlorides in a pseudo-equatorial fashion to face the two consecutive guanine residues of the 25S rRNA (G2793 and G2794) (3). To address this question experimentally we solved the crystal structure of a C45/80S ribosome complex at 3.1 Å resolution. This allowed us to unravel the molecular interactions that C45 establishes with its target. We observed that C45 binds to the same binding pocket in the E-site of the LSU as CL, where the tRNA CCA-end should position during the elongation phase. The superposition of CL and C45 allowed us to observe that the hydroxysuccinimide moiety is sitting in a very similar manner for the two compounds, creating extensive interactions with residues G92, C93, U2763 and A2802 of the 25S rRNA. Additionally, C45’s C3 chlorine, in conjunction with the other axial substituents present in the A ring, helps to favor the adoption of a twist-boat conformation, thus establishing a second halogen–π interaction with G2793 (or G4370 in humans), as previously predicted (3). We noted that C45 does not interact directly with the eukaryotic specific eL42 ribosomal protein (as CL does) owing to the lack of the hydroxyl group that CL has in position C7 (Figure 1). We can therefore speculate that the loss of this specific H-bond might be responsible for some of the difference in activity compared to CL (3). Dispersion interaction studies of a minimized model of C45 and CL within the contacting rRNA residues of the 25S rRNA, in the attempt of providing rationale of all the intermolecular interactions participating in binding, confirmed our hypothesis (Table 2).

We then asked ourselves whether these differences in the binding mode of C45 would translate into a change of affinity toward the eukaryotic ribosome. To gain experimental insights and determine the affinity of lissoclimides to both human and yeast ribosomes, we performed smFRET imaging studies on *in vitro* assembled pre-translocation 80S ribosome complexes supplemented with the different E-site binding drugs (Figure 4). Our experimental results show that the dissociation constants for CHX and CL are only modestly different (1 and 4 versus 9.7 and 10 μM), in agreement with translation inhibition activities (3). The dissociation constant for hatQ, a congener of CL that lacks chloride substituents, but contains the hydroxyl group in position C7, is much larger, 100 and 400 μM (Figure 4). We can speculate that the difference in binding affinity might be due to the binding pose of hatQ in the E-site (Supplementary Figure S4). The absence of moderate to strong interactions between the decalin ring of hatQ and the 25S rRNA may destabilize the binding of the drug within the pocket. Finally, C45 has lower affinity than CL (apparent *K*_D_ of 9.7 and 10 μM) for the E-site binding pocket with dissociation constants of 26 and 37 μM, in agreement with translation inhibition assays performed for diverse lissoclimide congeners (3) as well as our dispersion interaction calculations. Taken together, our results suggest that the intermolecular interactions established by the second Cl atom within the 25S rRNA may compensate for the lack of hydrogen bonding to eL42 to further stabilize the bound inhibitor. As a consequence of our findings, we may look at halogen groups as a source of functionalization to improve pharmacodynamic characteristics of small-molecule inhibitors targeting the eukaryotic ribosome. This would permit engineering of different lissoclimide derivatives with similar binding properties achieved through distinct drug-target interactions.

**Figure 5.**
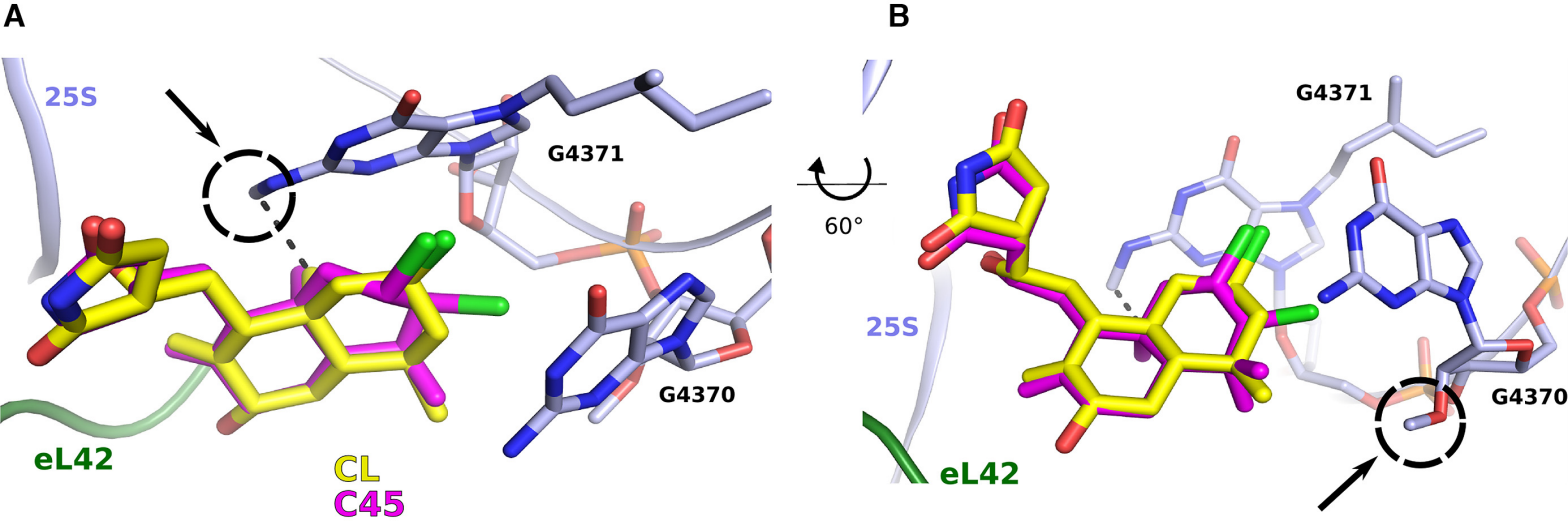
PTM on rRNA in humans can guide further drug-design. (**A, B**) Binding of CL (yellow) and C45 (magenta) within the E-site of the human ribosome. The proposed views (60 degrees apart) show how the lissoclimide might interact with the rRNA modifications recently modelled on the human 80S ribosome (PDB ID: 6EK0). In (A), the putative hydrophobic interaction between the C10 methyl group on both CL and C45 and the methylated G4371 on the 28S rRNA. By contrast, the 2′-O methylation of G4370 does not interact directly with the drugs but can be considered as target for the design of future compounds with higher potency. PTM modifications are circled for clear identification. CL is represented in yellow, while C45 in magenta.

We finally compared our structure with the cryo-EM structure of the *Homo sapiens* 80S ribosome (32) (PDB ID: 6EK0); superposition of the minimized E-site regions of the 25S and 28S rRNA yielded a root-mean square deviation (RMSD) value of 0.685 Å, and provided additional information about the expected interactions of C45 within the mammalian E-site binding pocket (Figure 5). Particularly interesting are potential drug interactions with post-translational modifications (PTM) of 28S rRNA residues within the E-site (more specifically on G4370 and G4371, corresponding to yeast G2793 and G2794) that might reflect the difference in binding affinities we observed for yeast and human ribosomes (32,33). For instance, lissoclimides may have the potential to establish a hydrophobic interaction between the methyl group on C10 of the decalin ring with the methyl moiety of m^2^xp^7^G4371 (Figure 5). Lissoclimides may also be derivatized to reach the nearby ribose 2′-*O*-methylation on residue G4370. Increasing evidence that rRNA modifications are not constitutively expressed onto the ribosome (33), and recent evidence for pervasive sequence variation in rRNA with tissue specific expression (34) adds an additional layer of complexity and calls for efforts for the design of more potent and, highly specific, anti-cancer drugs to combat resistance development and target heterogeneity.

## DATA AVAILABILITY

Atomic coordinates and structure factors for the reported crystal structures have been deposited with the Protein Data bank under accession number 6HHQ.

## Supplementary Material

Supplementary Data
