# Supplementary material for: Understanding the role of intermolecular interactions between lissoclimides and the eukaryotic ribosome
Source: Nucleic Acids Res. 2019 Feb 13;47(6):3223–32. doi: 10.1093/nar/gkz053 (PMC6451132; doi:10.1093/nar/gkz053)
Supplement: Supplementary Data [file gkz053_supplemental_file.pdf]

SUPPLEMENTARY DATA

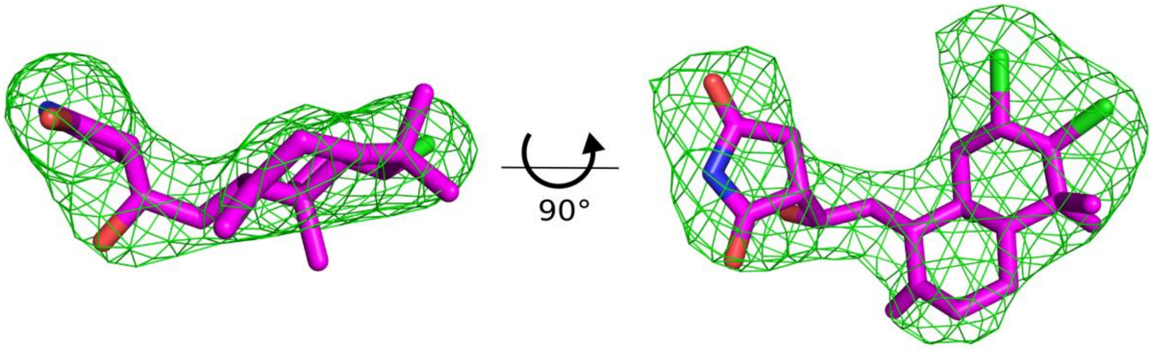

Figure S1. Unbiased difference density helped us to fit C45 unambiguously. $F_{obs} - F_{calc}$ density map obtained after the first run of rigid-body refinement, using the vacant yeast 80S ribosome structure (PDB ID: 4V88) as reference model. Density is shown in green, contoured at 3σ and represented as two views, 90° apart.

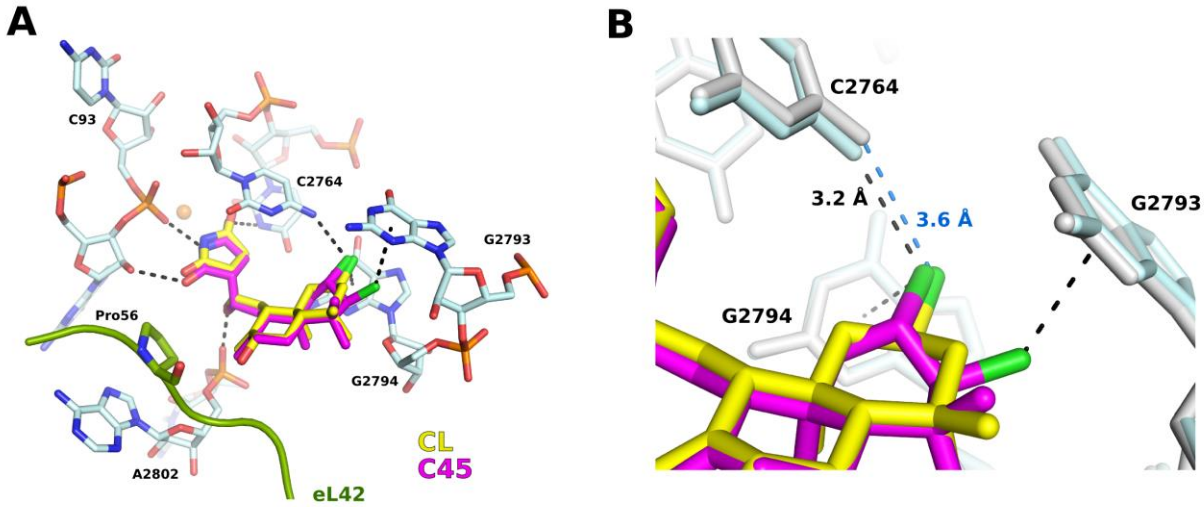

Figure S2. Comparison of the binding mode of C45, CL and hatQ within the E-site binding pocket. (A) The binding mode of the two lissoclimides is overall very similar. (B) Zoom-in into the pocket showing the differences in terms of the interaction network of the decalin ring, which bears the halogen groups. The superposition of the two binding pockets from CL/80S and C45/80S structures

resulted in an RMSD of 0.187 Å. 25S rRNA from CL/80S complex is shown in grey, while 25S rRNA of C45/80S complex in cyan.

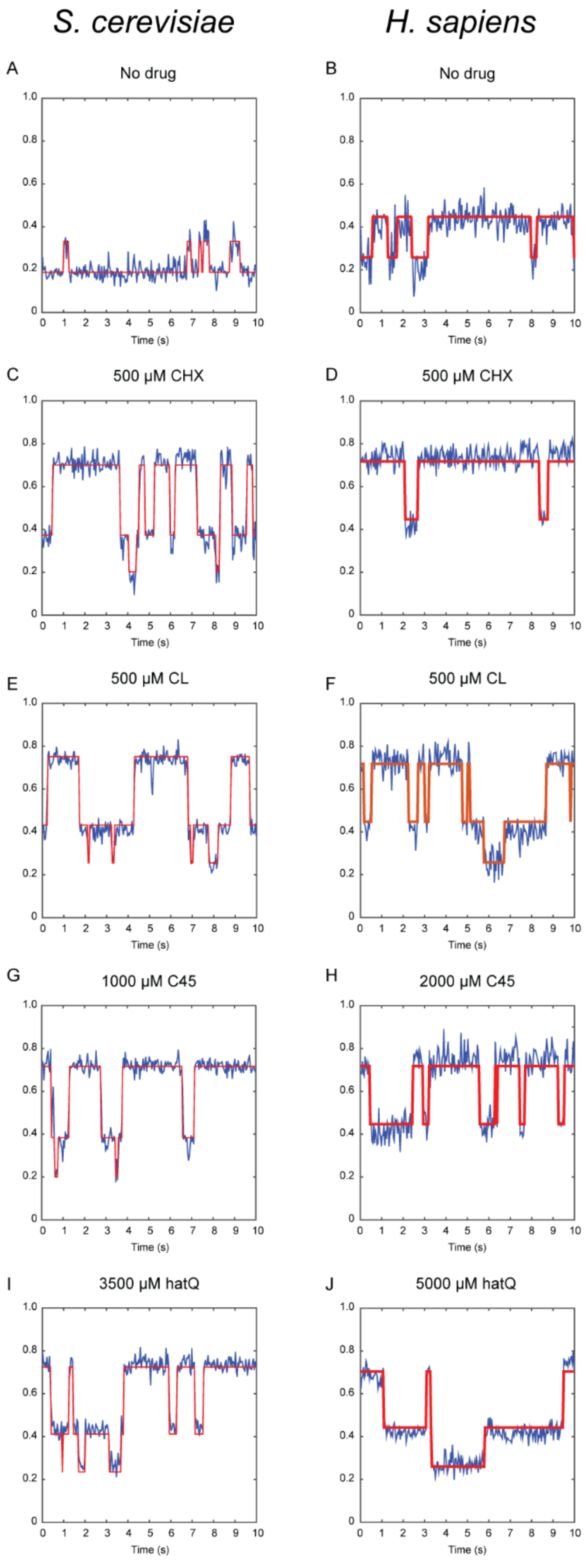

Figure S3. Single-molecule FRET traces illustrating excursions into lower-FRET, hybrid-like states on the yeast and human pre-translocation ribosome complex at saturating or near-saturating

drug concentrations. (A/B) Time-dependent FRET efficiency calculated from Cy3 and Cy5 fluorescence intensities from a single yeast and human pre-translocation ribosomes in the absence of drug. The blue line is the estimated FRET efficiency and the solid red line represents hidden Markov model-based idealization of the data. In the absence of E-site binding drugs the pre-translocation ribosome complex predominantly occupies, and fluctuates between, the two hybrid tRNA states. Time-dependent smFRET efficiency of representative molecules in which excursions into hybrid-like FRET states are readily apparent (C/D) at 500 µM CHX, (E/F) at 500 µM CL, (G/H) at 1000 and 2000 µM C45, (I/J) at 3500 and 5000 µM hatQ.

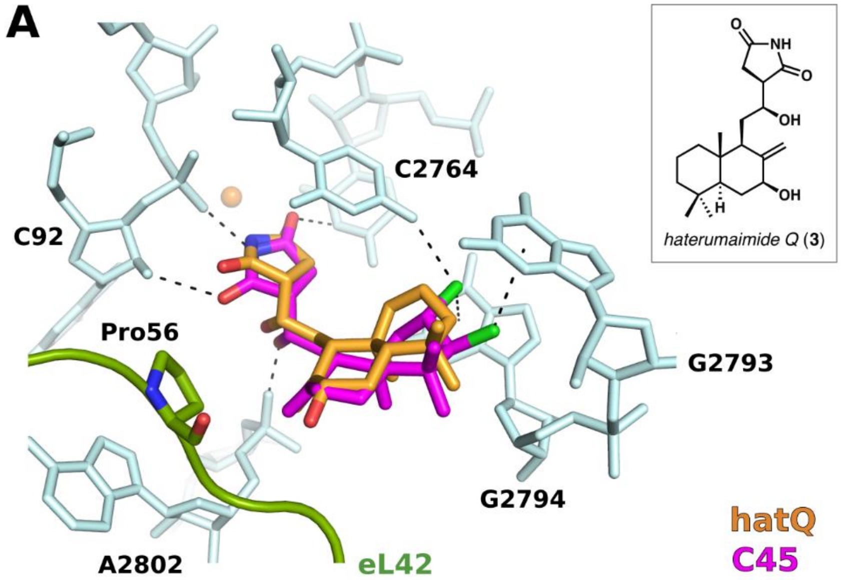

Figure S4. hatQ binds to the E-site pocket with lower affinity than C45. (A) Binding pose of hatQ (orange) within the E-site pocket of the LSU. The pose is predicted by molecular docking, as determined previously (3). Here we can observe how the decalin ring is misplaced compared to C45 (magenta). For clarity, only the 25S rRNA of the experimentally determined C45/80S complex (cyan) is shown. hatQ chemical structure is shown for clarity.
